# Serum uric acid levels and the risk of hemorrhagic stroke: Insights from a two-sample Mendelian randomization study

**DOI:** 10.1016/j.clinsp.2025.100726

**Published:** 2025-07-30

**Authors:** Zi-Wen Wang, Fang Zhao, Jin-Chao Liu, Dan-Feng Li

**Affiliations:** a Department of Interventional Radiology, Puyang Oilfield General Hospital, Puyang, Henan Province, China; b Department of Sterilization Supply Center, Puyang Oilfield General Hospital, Puyang, Henan Province, China

**Keywords:** Mendelian randomization, Serum uric acid, Intracerebral hemorrhage, Subarachnoid Hemorrhage, Causal Inference

## Abstract

**Background and aims:**

Observational studies have indicated a potential association between serum uric acid levels and the risk of intracerebral hemorrhage and subarachnoid hemorrhage. To explore whether this relationship is causal, the authors performed a two-sample Mendelian randomization analysis.

**Methods and results:**

The authors sourced data on single-nucleotide polymorphisms related to serum uric acid levels from the European Bioinformatics Institute ‒ Association database, along with information on intracerebral hemorrhage and subarachnoid hemorrhage from the same repository. The primary analysis for the Mendelian Randomization was conducted using the inverse variance weighted method, accompanied by sensitivity analyses to validate the findings. The Mendelian Randomization analysis revealed a positive causal association between serum uric acid and the risk of ICH Odds Ratio (OR = 1.29; 95 % CI 1.07–1.54, *p* = 0.007) and SAH (OR = 1.27; 95 % CI 1.04–1.54, *p* = 0.018).

**Conclusion:**

The present research offers direct evidence suggesting that higher serum uric acid levels could raise the risk of Intracerebral Hemorrhage (ICH) and Subarachnoid Hemorrhage (SAH). Additional investigations are needed to explore the mechanisms by which serum uric acid influences these types of hemorrhagic events.

## Highlight

• Study explores uric acid's impact on hemorrhagic stroke risk.
• Suggests uric acid contributes to endothelial dysfunction and stress.
• Findings enhance understanding of uric acid and stroke mechanisms.
• Aims to clarify biological pathways linking uric acid and stroke.

## Introduction

Hemorrhagic stroke is a critical neurological disorder characterized by the rupture of blood vessels, resulting in blood infiltration into brain tissue. This condition is classified into various subtypes based on its location and etiology, including spontaneous intracerebral hemorrhage, traumatic brain hemorrhage, and aneurysmal rupture. Epidemiological data indicate a rising incidence of hemorrhagic strokes globally, particularly among the elderly, highlighting a robust association between age and the risk of occurrence.^1^ This escalating prevalence not only severely impairs patients' quality of life but also imposes substantial burdens on healthcare systems and economies. Common risk factors for hemorrhagic stroke include hypertension, diabetes, smoking, and alcohol consumption, yet several other potential risk factors warrant further investigation.^2^

In recent years, Serum Uric Acid (SUA) has emerged as an important biomarker, prompting extensive research into its relationship with cerebrovascular diseases.^3^ Some studies suggest that elevated SUA levels may be associated with the risk of hemorrhagic stroke.^4^, ^5^, ^6^ Conversely, opinions in this field remain inconsistent. Some research indicates that hyperuricemia may increase the risk of hemorrhagic stroke by promoting oxidative stress and inflammatory responses, while other studies propose that uric acid, as an antioxidant, may have a protective role.^7^ Therefore, while some observational studies have hinted at a correlation between SUA and hemorrhagic stroke^8^ there is still a lack of sufficient causal evidence to validate this hypothesis. Moreover, various confounding factors and the possibility of reverse causation add complexity to this matter.

Mendelian Randomization (MR) research^9^ which relies on genetic variation, provides a novel method for exploring the association between SUA levels and the risk of hemorrhagic stroke. This approach reduces confounding bias and the possibility of reverse causality by leveraging the independence between genetic variants and environmental factors. Consequently, it enhances the capacity for causal inference.^10^, ^11^, ^12^ This study aims to employ MR to thoroughly examine the association between SUA levels and the risk of hemorrhagic stroke. The goal is to offer scientific insights that enhance the understanding of the underlying mechanisms and to inform risk prediction and intervention strategies.

## Methods

### Study design

This study aimed to explore the causal relationship between SUA levels and the risk of hemorrhagic stroke, focusing specifically on Intracerebral Hemorrhage (ICH) and Subarachnoid Hemorrhage (SAH), using a two-sample MR approach. In this analysis, SUA served as the exposure variable, while the occurrence of hemorrhagic stroke was regarded as the outcome. To conduct a valid MR study, three key assumptions must be satisfied: first, the chosen SNPs must exhibit a significant association with SUA levels; second, these SNPs should be free from any confounding influences; and third, the SNPs should affect hemorrhagic stroke solely through their impact on SUA levels. While this MR study was not pre-registered, the analytical workflow strictly adhered to established MR guidelines using publicly available GWAS summary statistics, ensuring transparency in hypothesis testing. For future studies involving novel hypotheses or primary data collection, the authors will implement pre-registration protocols to further strengthen methodological rigor.

### Ethical statement

The current analysis did not require additional ethics approval because all Genome-Wide Association Study (GWAS) data used in this research are publicly available and were originally collected under studies that had obtained approval from their respective ethical review committees.

### Data resources

Genome-Wide Association Studies (GWAS) summary statistics for SUA were obtained from a cross-population atlas of genetic associations that includes 220 human phenotypes, based on data from as many as 343,836 individuals of European ancestry. ^13^ Data for intracerebral hemorrhage, including 1935 cases and 473,513 controls, and for subarachnoid hemorrhage, with 1693 cases and 473,255 controls, were obtained from the European Bioinformatics Institute ‒ Association (EBI-A). EBI-A is a comprehensive database that collects and organizes Genome-Wide Association Study (GWAS) results related to various diseases and traits. It provides easy access to information on genetic variants, effect sizes, and sample characteristics, enabling researchers to explore the genetic factors underlying health conditions. Regular updates ensure users have access to the latest genetic research findings.^14^ The specifics are outlined in Table 1.

**Table 1:** Characteristics of data sources and strength of IVs used in the Mendelian randomization study.

Exposures/ Outcomes	Year	Author	Consortium	Ethnicity	Sample Sizes	Number of SNPs	F-Statistic (Average)
Serum uric acid	2021	Sakaue S	EBI-A	European	343.836	19.041.286	NA
ICH	2021	Sakaue S	EBI-A	European	473.513	24.191.284	66,859
SAH	2021	Sakaue S	EBI-A	European	473.255	24.191.735	62,267

ICH, Intracerebral Hemorrhage; SAH, Subarachnoid Hemorrhage; SNPs, Single Nucleotide Polymorphisms; NA, Not Available.

### Selection of genetic instrumental variables

Genetic variants that showed a significant association with SUA at the genome-wide level (*p* < 5 × 10^–8^) were selected as instrumental variables. Additionally, independent SNPs were selected to minimize the impact of linkage disequilibrium, defined by r² < 0.001 and a clumping window of 10,000 kb. SNPs related to potential confounders were subsequently excluded. In this study, hypertension, smoking, Body Mass Index (BMI), and arteriosclerosis were recognized as confounding factors (http://www.phenoscanner.medschl.cam.ac.uk/).^15^ SNP harmonization was performed to align the orientation of the alleles.^16^ The F statistic for SNPs was utilized to identify those with a robust association between Instrumental Variables (IVs) and exposure factors. The equation employed is *F* = *R*² (N-K-1)/[K (1-R²)] where R² signifies the total explained variance attributed to the selected SNP in relation to the exposure, N denotes the sample size of the exposed dataset, and K represents the count of SNPs incorporated in the ultimate analysis. The F statistics for each instrument-exposure effect varied between 10.066 and 4333.022, indicating a minimal probability of weak instrumental bias (Table 1).

### Statistical analysis

In this MR analysis, the primary analytical strategy utilized was the Inverse Variance Weighted (IVW) method, which was applied to estimate the causal associations between various exposures and their corresponding outcomes.^17^ The variation among the estimates of genetic variants was evaluated utilizing Cochran's *Q* test. In instances where the p-value obtained from this test was below 0.05, a random-effects model was applied for the IVW analysis; however, if the p-value was equal to or greater than 0.05, a fixed-effects model was adopted.^18^ Supplementary analyses included the weighted median method,^19^ Simple mode, MR-Egger regression,^20^ and the weighted mode, all used alongside IVW.

### Sensitivity analysis

The MR-Egger test was conducted to assess the possibility of pleiotropy, revealing a p-value for the MR-Egger intercept greater than 0.05. This indicates a lack of horizontal pleiotropy. To examine the robustness of the results, sensitivity analyses were carried out using a leave-one-out approach, which involved removing each SNP one at a time. Furthermore, funnel plots and forest plots were created to assess the potential existence of pleiotropy. A significance level of *p* < 0.05 was established for statistical relevance. The analyses were performed utilizing the “Two-Sample-MR” package within R software, version 4.4.1. It is important to highlight that the study protocol and its associated details were not pre-registered in an online database.

## Results

### IVs selection

The details of the IVs for SUA used in the MR analysis are presented in Supplementary Tables 1 and 2. Ultimately, the authors employed 135 SNPs as instrumental variables for the association between SUA and ICH (Supplementary Table 1) and 135 SNPs for SAH (Supplementary Table 2).

### Effects of SUA on hemorrhagic stroke

**Fig. 1:**
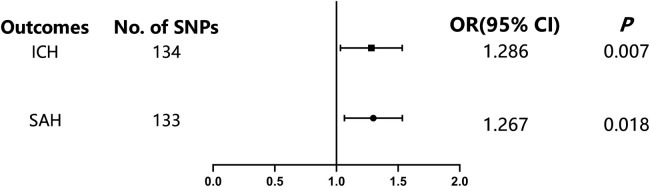
Associations of genetically predicted serum uric acid with lower limb vascular diseases. CI, Confidence Interval; OR, Odds Ratio; SNP, Single-Nucleotide Polymorphism; SAH, Subarachnoid Hemorrhage; ICH, Intracerebral Hemorrhage.

Table 2 demonstrates a significant correlation between SUA levels and the occurrence of hemorrhagic stroke. Utilizing the random model IVW method, the present analysis indicated that elevated levels of SUA are correlated with a heightened risk of ICH (OR = 1.29; 95 % CI 1.07‒1.54, *p* = 0.007) and SAH (OR = 1.27; 95 % CI 1.04‒1.54, *p* = 0.018) (Fig. 1). Other methods, including the weighted median method, MR-Egger method, simple mode, and weighted mode, also showed directionally similar estimates for ICH and SAH risks associated with SUA. Conversely, these methods were not sufficiently powered to reach conventional significance thresholds (Table 1). Specifically, the weighted median method found (OR = 1.18; 95 % CI 0.88‒1.59, *p* = 0.27) for ICH and (OR = 1.21; 95 % CI 0.85‒1.71, *p* = 0.30) for SAH. The MR-Egger method yielded (OR = 1.05; 95 % CI 0.80‒1.39, *p* = 0.72) for ICH and (OR = 1.09; 95 % CI 0.79‒1.49, *p* = 0.61) for SAH. The simple mode method found (OR = 1.36; 95 % CI 0.71‒2.60, *p* = 0.35) for ICH and (OR = 1.18; 95 % CI 0.58‒2.37, *p* = 0.65) for SAH. Finally, the weighted mode method found (OR = 1.26; 95 % CI 0.98‒1.61, *p* = 0.07) for ICH and (OR = 1.21; 95 % CI 0.91‒1.612, *p* = 0.20) for SAH.

**Table 2:** Mendelian randomization for statin on the risk of ICH and SAH.

	ICH			SAH		
	OR Estimate (95 % CI)	p-value	Beta	OR Estimate (95 % CI)	p-value	Beta
**MR Egger**	1.051 (0.797‒1.387)	0.724	0.050	1.085(0.791‒1.490)	0.613	0.082
**Weighted median**	1.182 (0.878‒1.590)	0.271	0.167	1.205 (0.850‒1.710)	0.296	0.186
**IVW (random effects)**	1.286 (1.072‒1.541)	0.007	0.251	1.267 (1.041‒1.542)	0.018	0.237
**Simple mode**	1.362 (0.713‒2.601)	0.352	0.309	1.175 (0.582‒2.374)	0.654	0.161
**Weighted mode**	1.257 (0.980‒1.613)	0.074	0.223	1.210 (0.908‒1.612)	0.196	0.191

IVW, Inverse Variance Weighted; OR, Odds Ratio; ICH, Intracerebral Hemorrhage; SAH, Subarachnoid Hemorrhage.

### Sensitivity analyses for MR analysis

Sensitivity analyses confirmed the robustness of the present findings. MR-Egger regression revealed no significant horizontal pleiotropy (intercept *p* > 0.05; Table 3), and Cochran’s *Q* test indicated minimal heterogeneity (*p* > 0.05). Leave-one-out analysis demonstrated that no single SNP disproportionately influenced the causal estimates (Supplementary Fig. 4 A‒B). Notably, rs45499402 showed a marginal outlier effect for SAH; its exclusion did not alter the overall significance (IVW: OR = 1.26, 95 % CI 1.03–1.54, *p* = 0.024). Scatter, forest, and funnel plots are provided in Supplementary Figures 1–3.

**Table 3:** Pleiotropy and heterogeneity test for serum uric acid on ICH and SAH.

	Pleiotropy test			Heterogeneity test					
	MR-Egger			MR-Egger			Inverse variance weighted		
	Intercept	SE	P	Q	Q_df	Q_pval	Q	Q_df	Q_pval
**ICH**	0.009	0.005	0.064	139.501	127	0.211	143.336	128	0.168
**SAH**	0.006	0.005	0.224	124.952	126	0.510	126.442	127	0.497

ICH, Intracerebral Hemorrhage; SAH, Subarachnoid Hemorrhage; SNPs, Single Nucleotide Polymorphisms.

## Discussion

SUA has been increasingly recognized as a significant biomarker and potential contributor to cerebrovascular diseases, including hemorrhagic stroke. Elevated SUA levels have been associated with an increased risk of hemorrhagic stroke through multiple mechanisms, including endothelial dysfunction, oxidative stress, and inflammation. Conversely, the role of uric acid in vascular health is complex and dual, as it can exhibit both protective and harmful effects depending on the context.

SUA exhibits context-dependent dual effects: while its antioxidant properties may transiently mitigate oxidative stress in acute ischemic conditions,^28^^,^^30^ chronic hyperuricemia predominantly promotes endothelial dysfunction and vascular inflammation. Elevated SUA reduces nitric oxide bioavailability via eNOS uncoupling, exacerbating hypertension,^31^^,^^39^ while simultaneously activating pro-inflammatory pathways (e.g., NLRP3/NF-κB) that weaken vascular integrity.^34^^,^^40^ This paradox may explain observational studies suggesting protective roles of SUA in ischemic stroke versus the findings of harm in hemorrhagic subtypes. The net effect likely hinges on exposure duration, metabolic context (e.g., coexisting hypertension), and genetic factors influencing SUA distribution.^24^^,^^27^ The present results emphasize that chronic hyperuricemia tilts the balance toward harmful vascular remodeling, outweighing transient antioxidant benefits.

These findings corroborate previous studies investigating the relationship between SUA levels and various cerebrovascular conditions. Several studies, such as those by Zhang et al.,^21^ and Diallo et al.,^22^ suggest that high uric acid levels can lead to endothelial dysfunction and increased oxidative stress, worsening vascular injuries, and potentially causing hemorrhagic events.^23^ Zhang et al.,^21^ for example, discovered that high uric acid levels are linked to reduced nitric oxide availability and higher oxidative stress markers in patients, suggesting a mechanism by which uric acid could harm vascular health.^24^, ^25^, ^26^ In contrast, Zhang et al.,^27^ Noted that uric acid might protect against stroke by acting as an antioxidant, potentially decreasing neuroinflammation and neuronal damage during acute ischemic events.^28^, ^29^, ^30^

Increased SUA levels may affect the risk of ICH and SAH through various interconnected molecular mechanisms. A key pathway involves the induction of endothelial dysfunction, characterized by decreased Nitric Oxide (NO) production and increased oxidative stress. This dysfunction can weaken blood vessel walls, making them more susceptible to rupture.^31^, ^32^, ^33^ Additionally, hyperuricemia can promote vascular inflammation by activating inflammatory mediators and transcription factors like Nuclear Factor kappa B (NF-κB),^34^ leading to structural changes in blood vessels that compromise their integrity. Elevated uric acid can also induce smooth muscle cell proliferation and migration,^35^ contributing to vascular remodeling and further weakening blood vessel structure.^36^, ^37^, ^38^ Notably, high SUA levels are often associated with other cardiovascular risk factors, particularly hypertension, as uric acid can cause vasoconstriction and interfere with blood pressure regulation.^39^ This exacerbation of hypertension, along with oxidative stress-induced damage to endothelial cells and the extracellular matrix, ultimately enhances the risk of hemorrhagic events in the brain.^40^ In summary, the relationship between SUA and ICH or SAH risk is complex, involving endothelial dysfunction, vascular inflammation, and interactions with essential cardiovascular factors, underscoring potential therapeutic targets for mitigating ICH/SAH risk.

Confounding factors such as hypertension, diabetes, and smoking may bias observational studies investigating the relationship between SUA and hemorrhagic stroke. MR addresses this limitation by leveraging genetic instrumental variables associated with SUA levels but independent of environmental or behavioral factors, thereby minimizing confounding bias. Unlike traditional observational studies, MR allows researchers to more reliably evaluate the independent causal role of SUA in hemorrhagic stroke.

Conversely, this study is not without limitations. The current findings, primarily derived from European populations, may have limited generalizability due to racial and ethnic variations in genetic backgrounds, environmental exposures, and SUA metabolism. For instance, African American populations frequently demonstrate elevated SUA levels and distinct metabolic patterns compared to European cohorts. To strengthen the global applicability of these results and clarify SUA’s role in hemorrhagic stroke across diverse demographics, future studies should rigorously validate these associations in non-European populations. Such efforts would advance understanding of population-specific mechanisms underlying SUA-related stroke risk. Additionally, there remains a possibility of horizontal pleiotropy, as the authors only excluded SNPs associated with known confounders. Future studies should aim to replicate these findings in diverse populations to fully understand the relationship between SUA and intracerebral hemorrhage risk.

## Conclusion

In conclusion, the authors employed a two-sample MR approach to provide genetic evidence suggesting that higher SUA levels may elevate the risk of ICH and SAH. Additional research is necessary to validate the harmful effects of increased SUA on ICH and SAH. Large-scale randomized controlled trials should also be conducted to confirm the present findings from the MR analysis.

## Availability of data and material

All the data utilized in this study were publicly accessible.

## Authors’ contribution

ZW.W conceived the idea, designed the study protocol, and revised the manuscript. ZF and JC.L were responsible for data collection and analysis. DF.L contributed to the writing of the manuscript. All authors reviewed and approved the final version of the manuscript.

## Conflicts of interest

The authors declare that this research was carried out without any commercial or financial relationships that could be perceived as a potential conflict of interest.

## Appendix Supplementary materials

Image, application 1

Image, application 2

Image, application 3

Image, application 4

Image, application 5

Image, application 6
