# Supplementary material for: Serum uric acid levels and the risk of hemorrhagic stroke: Insights from a two-sample Mendelian randomization study
Source: Clinics (Sao Paulo). 2025 Jul 30;80:100726. doi: 10.1016/j.clinsp.2025.100726 (PMC12332956; doi:10.1016/j.clinsp.2025.100726)
Supplement: Supplementary file 1 [file mmc1.docx]

CLINICS-D-24-01435_Supplementary Material

**Figure S1** Scatter plot of MR effect size for causal associations. (A) Serum uric acid and intracerebral hemorrhage. (B) Serum uric acid and subarachnoid hemorrhage.

**Figure S2** Forest plot of MR effect size using MR-Egger and IVW methods for causal associations (A) Serum uric acid and intracerebral hemorrhage. (B) Serum uric acid and subarachnoid hemorrhage.

**Figure S3** Funnel plot of causal associations. (A) Serum uric acid and intracerebral hemorrhage. (B) Serum uric acid and subarachnoid hemorrhage

**Figure S4** Leave-one-out plot to assess if a single variant is driving the association. (A) Serum uric acid and intracerebral hemorrhage. (B) Serum uric acid and subarachnoid hemorrhage.
