# Supplementary material for: Serum uric acid levels and the risk of hemorrhagic stroke: Insights from a two-sample Mendelian randomization study
Source: Clinics (Sao Paulo). 2025 Jul 30;80:100726. doi: 10.1016/j.clinsp.2025.100726 (PMC12332956; doi:10.1016/j.clinsp.2025.100726)
Supplement: Supplementary file 4 [file mmc4.docx]

Figure 1 Associations of genetically predicted serum uric acid with lower limb vascular diseases


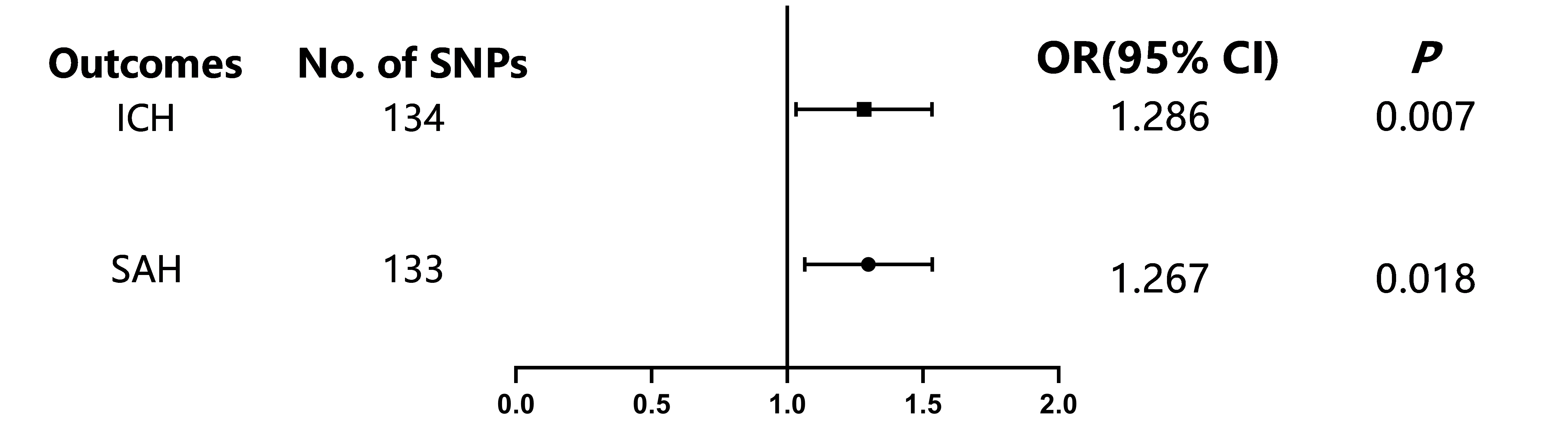


CI, confidence interval; OR, odds ratio; SNP, single-nucleotide polymorphism; SAH, subarachnoid hemorrhage; ICH：intracerebral hemorrhage
