# Supplementary material for: Serum uric acid levels and the risk of hemorrhagic stroke: Insights from a two-sample Mendelian randomization study
Source: Clinics (Sao Paulo). 2025 Jul 30;80:100726. doi: 10.1016/j.clinsp.2025.100726 (PMC12332956; doi:10.1016/j.clinsp.2025.100726)
Supplement: Supplementary file 5 [file mmc5.docx]

**STROBE-MR checklist of recommended items to address in reports of Mendelian randomization studies**^1^ ^2^

| **Item No.** | **Section** | **Checklist item** | **Page No.** | **Relevant text from manuscript** |
| --- | --- | --- | --- | --- |
| 1 | **TITLE and ABSTRACT** | Indicate Mendelian randomization (MR) as the study’s design in the title and/or the abstract if that is a main purpose of the study | 1 | Serum Uric Acid Levels and Risk of Hemorrhagic Stroke: A Two-Sample Mendelian Randomization Study |
|  | **INTRODUCTION** |  |  |  |
| 2 | **Background** | Explain the scientific background and rationale for the reported study. What is the exposure? Is a potential causal relationship between exposure and outcome plausible? Justify why MR is a helpful method to address the study question | 2.3.4. | Hemorrhagic stroke is a critical neurological disorder characterized by the rupture of blood vessels, resulting in blood infiltration into brain tissue....serum uric acid has emerged as an important biomarker, prompting extensive research into its relationship with cerebrovascular diseases...Mendelian randomization (MR) research, a method based on genetic variation, offers a unique approach for investigating the relationship between serum uric acid and the risk of hemorrhagic stroke... |
| 3 | **Objectives** | State specific objectives clearly, including pre-specified causal hypotheses (if any). State that MR is a method that, under specific assumptions, intends to estimate causal effects | 4.5. | This study aims to employ Mendelian randomization to systematically evaluate the association between serum uric acid levels and hemorrhagic stroke risk, intending to provide scientific evidence for a deeper understanding of underlying mechanisms and guidance for risk prediction and intervention strategies. |
|  | **METHODS** |  |  |  |
| 4 | **Study design and data sources** | Present key elements of the study design early in the article. Consider including a table listing sources of data for all phases of the study. For each data source contributing to the analysis, describe the following: |  |  |
|  | a) | Setting: Describe the study design and the underlying population, if possible. Describe the setting, locations, and relevant dates, including periods of recruitment, exposure, follow-up, and data collection, when available. | 4.5. | This study sought to examine the causal link between serum uric acid and the risk of hemorrhagic stroke, including intracerebral hemorrhage (ICH) and subarachnoid hemorrhage (SAH), using a two-sample Mendelian randomization (MR) method...Genome-Wide Association Studies (GWAS) summary statistics for serum uric acid were sourced from a cross-population atlas of genetic associations encompassing 220 human phenotypes, with data from up to 343,836 participants of European ancestry... |
|  | b) | Participants: Give the eligibility criteria, and the sources and methods of selection of participants. Report the sample size, and whether any power or sample size calculations were carried out prior to the main analysis | 5. | Data for intracerebral hemorrhage, including 1935 cases and 473,513 controls, and for subarachnoid hemorrhage, with 1,693 cases and 473,255 controls... |
|  | c) | Describe measurement, quality control and selection of genetic variants | 5. | Genetic variants associated with serum uric acid at genome-wide significance (P < 5 ×10-8 ) were selected as instruments. Moreover, independent SNPs to avoid offsets caused by linkage disequilibrium... |
|  | d) | For each exposure, outcome, and other relevant variables, describe methods of assessment and diagnostic criteria for diseases | 5. | Data for intracerebral hemorrhage, including 1935 cases and 473,513 controls, and for subarachnoid hemorrhage, with 1,693 cases and 473,255 controls, were obtained from the European Bioinformatics Institute - Association (EBI-A)... |
|  | e) | Provide details of ethics committee approval and participant informed consent, if relevant | 4. | We used summary data from previously published studies, which adhered to participant consent protocols and received ethical approval... |
| 5 | **Assumptions** | Explicitly state the three core IV assumptions for the main analysis (relevance, independence and exclusion restriction) as well assumptions for any additional or sensitivity analysis | 4. | To validly perform a Mendelian randomization (MR) study, three essential assumptions must be met:... |
| 6 | **Statistical methods: main analysis** | Describe statistical methods and statistics used |  |  |
|  | a) | Describe how quantitative variables were handled in the analyses (i.e., scale, units, model) | 6. | In this Mendelian randomization (MR) analysis, the inverse variance weighted (IVW) method was employed as the primary analytical approach to estimate causal relationships between exposures and outcomes... |
|  | b) | Describe how genetic variants were handled in the analyses and, if applicable, how their weights were selected | 5. | Genetic variants associated with serum uric acid at genome-wide significance (P < 5 ×10-8 ) were selected as instruments. Moreover, independent SNPs to avoid offsets caused by linkage disequilibrium, defined by r2 < 0.001 and clumping window = 10,000 kb, were selected as instrumental variables... |
|  | c) | Describe the MR estimator (e.g. two-stage least squares, Wald ratio) and related statistics. Detail the included covariates and, in case of two-sample MR, whether the same covariate set was used for adjustment in the two samples | 6. | Heterogeneity among genetic variant estimates was assessed using Cochran’s Q test. If the p-value from Cochran’s Q test was below 0.05, the MR analysis used the random-effects model of IVW; otherwise, the fixed-effects model was applied.. |
|  | d) | Explain how missing data were addressed |  | Not applicable |
|  | e) | If applicable, indicate how multiple testing was addressed |  | Supplementary analyses included the weighted median method, Simple mode, MR-Egger regression, and the weighted mode, all used alongside IVW... |
| 7 | **Assessment of assumptions** | Describe any methods or prior knowledge used to assess the assumptions or justify their validity | 3. | Some studies suggest that elevated serum uric acid levels may be associated with the risk of hemorrhagic stroke.. |
| 8 | **Sensitivity analyses and additional analyses** | Describe any sensitivity analyses or additional analyses performed (e.g. comparison of effect estimates from different approaches, independent replication, bias analytic techniques, validation of instruments, simulations) | 6. | The MR-Egger test was conducted to assess potential pleiotropy, with a P value for the MR-Egger intercept greater than 0.05 indicating the absence of horizontal pleiotropy. Sensitivity analyses, using a leave-one-out approach... |
| 9 | **Software and pre-registration** |  |  |  |
|  | a) | Name statistical software and package(s), including version and settings used | 6.7. | All analyses were performed using the “Two-Sample-MR” package in R software, Version 4.4.1. |
|  | b) | State whether the study protocol and details were pre-registered (as well as when and where) | 7. | This study protocol and details were not pre-registered online. |
|  | **RESULTS** |  |  |  |
| 10 | **Descriptive data** |  |  |  |
|  | a) |  | 7. | The details of the IVs for serum uric acid in MR analysis were represented in Supplementary Tables 1-2. Finally, we used 135 SNPs and 135 SNPs as instrument variables for serum uric acid on ICH (Supplementary Table 1) and SAH (Supplementary Table 2), respectively. |
|  | b) | Report summary statistics for phenotypic exposure(s), outcome(s), and other relevant variables (e.g. means, SDs, proportions) | 19. | Table 1 :Characteristics of data sources and strength of IVs used in the Mendelian randomization study. |
|  | c) | If the data sources include meta-analyses of previous studies, provide the assessments of heterogeneity across these studies |  | Not applicable |
|  | d) | For two-sample MR:  i.  Provide justification of the similarity of the genetic variant-exposure associations between the exposure and outcome samples  ii.  Provide information on the number of individuals who overlap between the exposure and outcome studies | 19. | Table 1 :Characteristics of data sources and strength of IVs used in the Mendelian randomization study. |
| 11 | **Main results** |  |  |  |
|  | a) | Report the associations between genetic variant and exposure, and between genetic variant and outcome, preferably on an interpretable scale | 7. | Table 2 indicates a positive association between serum uric acid and hemorrhagic stroke. Specifically, using the random model IVW, we observed that higher serum uric acid levels were associated with increased risks of ICH |
|  | b) | Report MR estimates of the relationship between exposure and outcome, and the measures of uncertainty from the MR analysis, on an interpretable scale, such as odds ratio or relative risk per SD difference | 7. | Table 2 indicates a positive association between serum uric acid and hemorrhagic stroke. Specifically, using the random model IVW, we observed that higher serum uric acid levels were associated with increased risks of ICH |
|  | c) | If relevant, consider translating estimates of relative risk into absolute risk for a meaningful time period |  | Not applicable |
|  | d) | Consider plots to visualize results (e.g. forest plot, scatterplot of associations between genetic variants and outcome versus between genetic variants and exposure) | 8. | The plots (scatter, forest, and funnel) for the analyses are shown in Supplementary Fig. 1A-B, 2A-B, and 3A-B. The leave-one-out analysis revealed that rs45499402 significantly influenced the causal estimate for SAH, leading us to exclude it from the instrumental variable selection for serum uric acid. |
| 12 | **Assessment of assumptions** |  |  |  |
|  | a) | Report the assessment of the validity of the assumptions | 8. | The MR-Egger intercepts provided no evidence of significant directional pleiotropy in the analysis results (Table 3). |
|  | b) | Report any additional statistics (e.g., assessments of heterogeneity across genetic variants, such as *I^2^*, Q statistic or E-value) | 8. | For the heterogeneity analysis, the Cochran's Q test showed no significant heterogeneity among SNPs associated with ICH and SAH (Table 3). |
| 13 | **Sensitivity analyses and additional analyses** |  |  |  |
|  | a) | Report any sensitivity analyses to assess the robustness of the main results to violations of the assumptions | 8. | The MR-Egger intercepts provided no evidence of significant directional pleiotropy in the analysis results (Table 3). For the heterogeneity analysis, the Cochran's Q test showed no significant heterogeneity among SNPs associated with ICH and SAH (Table 3). |
|  | b) | Report results from other sensitivity analyses or additional analyses |  | Not applicable |
|  | c) | Report any assessment of direction of causal relationship (e.g., bidirectional MR) | 7. | we observed that higher serum uric acid levels were associated with increased risks of ICH (OR: 1.286, 95% CI: 1.072-1.541, P = 0.007) and SAH (OR: 1.267, 95% CI: 1.041-1.542, P = 0.018). |
|  | d) | When relevant, report and compare with estimates from non-MR analyses | 7. | Other methods, including the weighted median method, MR-Egger method, simple mode, and weighted mode, also showed directionally similar estimates for ICH and SAH risks associated with serum uric acid. |
|  | e) | Consider additional plots to visualize results (e.g., leave-one-out analyses) | 7. | Supplementary Fig. 1-4 |
|  | **DISCUSSION** |  |  |  |
| 14 | **Key results** | Summarize key results with reference to study objectives | 8. | This study found that higher serum uric acid levels are causally linked to increased risks of ICH and SAH. Highlighting the importance of understanding its role in cerebrovascular diseases, these findings suggest that elevated serum uric... |
| 15 | **Limitations** | Discuss limitations of the study, taking into account the validity of the IV assumptions, other sources of potential bias, and imprecision. Discuss both direction and magnitude of any potential bias and any efforts to address them | 10. | However, this study is not without limitations. The dataset used in our analysis exhibited inherent heterogeneity... |
| 16 | **Interpretation** |  |  |  |
|  | a) | Meaning: Give a cautious overall interpretation of results in the context of their limitations and in comparison with other studies | 9. | Our findings corroborate previous studies investigating the relationship between serum uric acid levels and various cerebrovascular conditions. Several studies, such as those by Zhang et al. [33] and Diallo et al. [34], suggest that high uric acid levels can ... |
|  | b) | Mechanism: Discuss underlying biological mechanisms that could drive a potential causal relationship between the investigated exposure and the outcome, and whether the gene-environment equivalence assumption is reasonable. Use causal language carefully, clarifying that IV estimates may provide causal effects only under certain assumptions | 9. | Elevated serum uric acid levels may influence the risk of ICH/SAH through several interconnected molecular mechanisms. One primary pathway involves the induction ... |
|  | c) | Clinical relevance: Discuss whether the results have clinical or public policy relevance, and to what extent they inform effect sizes of possible interventions | 10. | In summary, the relationship between serum uric acid and ICH or SAH risk is complex, involving endothelial dysfunction, vascular inflammation, and interactions with essential cardiovascular factors, underscoring potential therapeutic targets for mitigating ICH/SAH risk... |
| 17 | **Generalizability** | Discuss the generalizability of the study results (a) to other populations, (b) across other exposure periods/timings, and (c) across other levels of exposure | 10. | Moreover, since our study population consisted exclusively of individuals of European descent, the applicability of these results to other ethnic groups remains uncertain. Future studies should aim to replicate these findings in diverse populations to fully understand the relationship between serum uric acid and intraintracerebral hemorrhage risk. |
|  | **OTHER INFORMATION** |  |  |  |
| 18 | **Funding** | Describe sources of funding and the role of funders in the present study and, if applicable, sources of funding for the databases and original study or studies on which the present study is based | 11. | This study was not supported by any funding. |
| 19 | **Data and data sharing** | Provide the data used to perform all analyses or report where and how the data can be accessed, and reference these sources in the article. Provide the statistical code needed to reproduce the results in the article, or report whether the code is publicly accessible and if so, where | 11. | All the data used in this study had been publicly available. |
| 20 | **Conflicts of Interest** | All authors should declare all potential conflicts of interest | 11. | The authors declare that the research was conducted in the absence of any commercial or financial relationships that could be construed as a potential conflict of interest. |

This checklist is copyrighted by the Equator Network under the Creative Commons Attribution 3.0 Unported (CC BY 3.0) license.

1. Skrivankova VW, Richmond RC, Woolf BAR, Yarmolinsky J, Davies NM, Swanson SA, et al. Strengthening the Reporting of Observational Studies in Epidemiology using Mendelian Randomization (STROBE-MR) Statement. JAMA. 2021;under review.

2. Skrivankova VW, Richmond RC, Woolf BAR, Davies NM, Swanson SA, VanderWeele TJ, et al. Strengthening the Reporting of Observational Studies in Epidemiology using Mendelian Randomisation (STROBE-MR): Explanation and Elaboration. BMJ. 2021;375:n2233.
