# Supplementary material for: Serum uric acid levels and the risk of hemorrhagic stroke: Insights from a two-sample Mendelian randomization study
Source: Clinics (Sao Paulo). 2025 Jul 30;80:100726. doi: 10.1016/j.clinsp.2025.100726 (PMC12332956; doi:10.1016/j.clinsp.2025.100726)

Supplementary materials

Figure S1 Scatter plot of MR effect size for causal associations

1. Serum uric acid and intracerebral hemorrhage


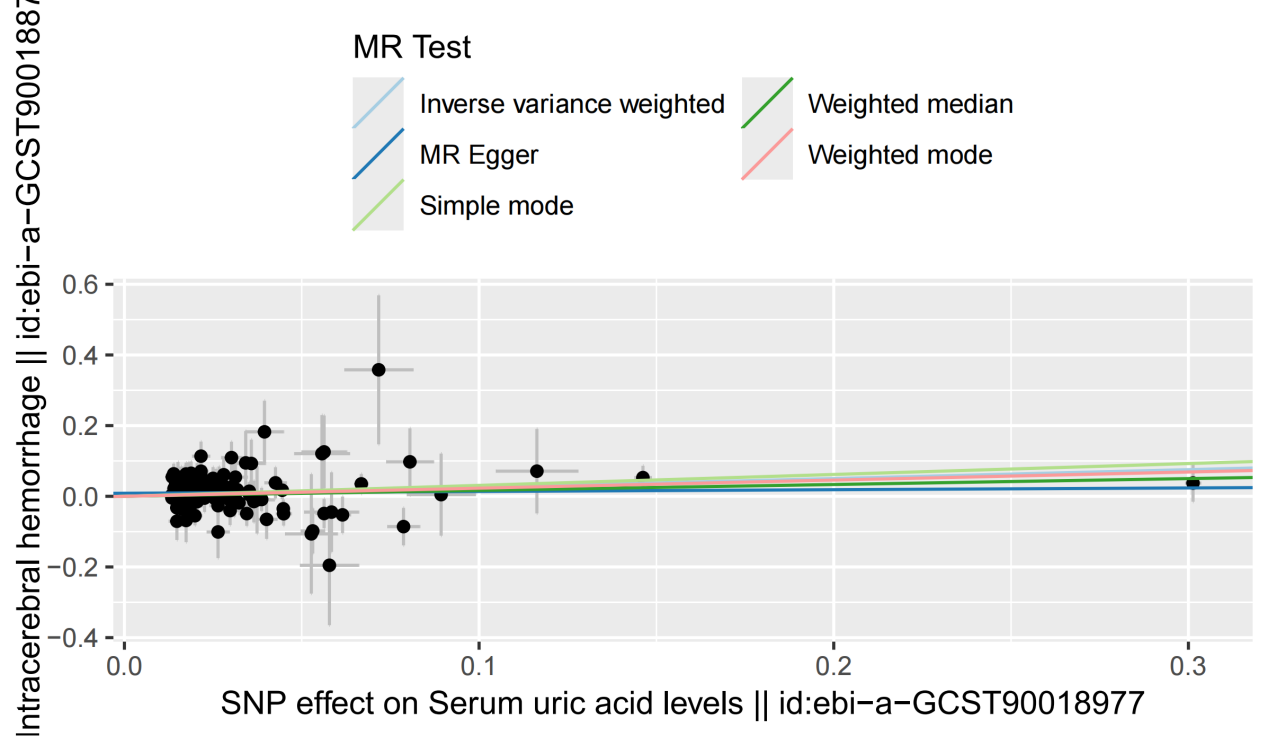


1. Serum uric acid and subarachnoid hemorrhage


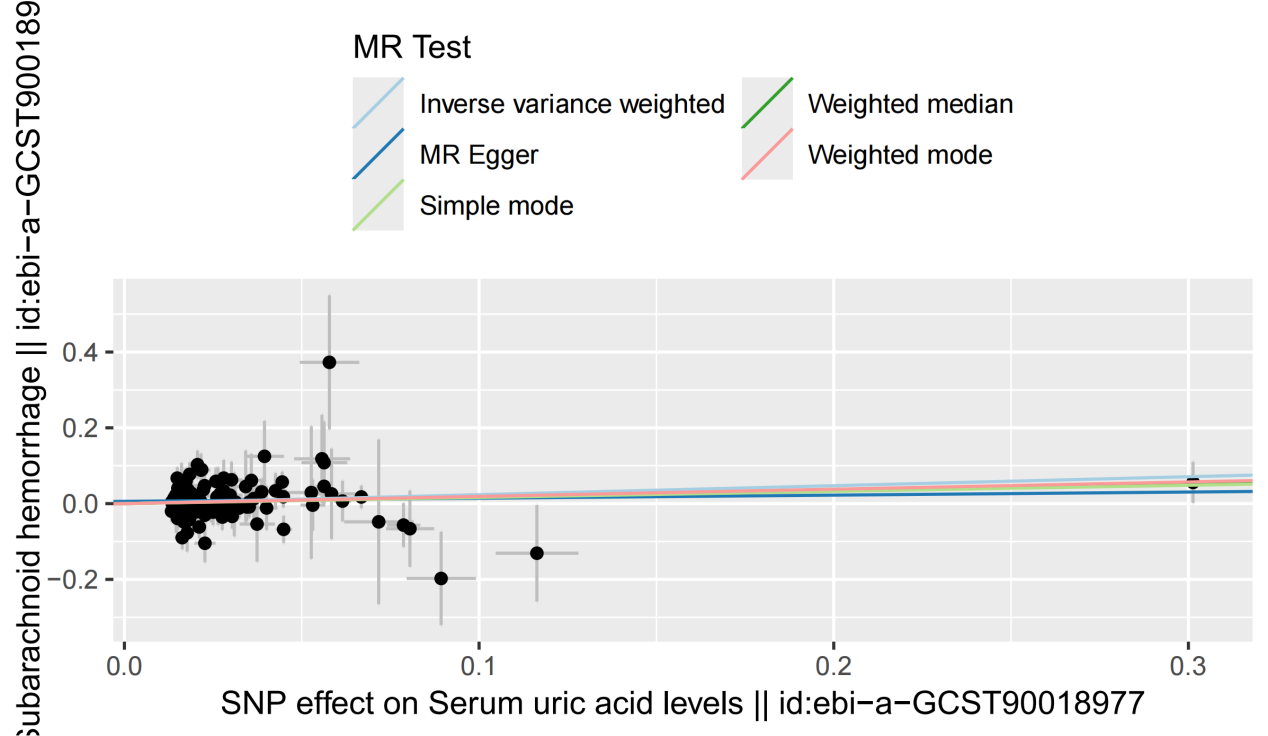


Figure S2 Forest plot of MR effect size using MR-Egger and IVW methods for causal associations

1. Serum uric acid and intracerebral hemorrhage


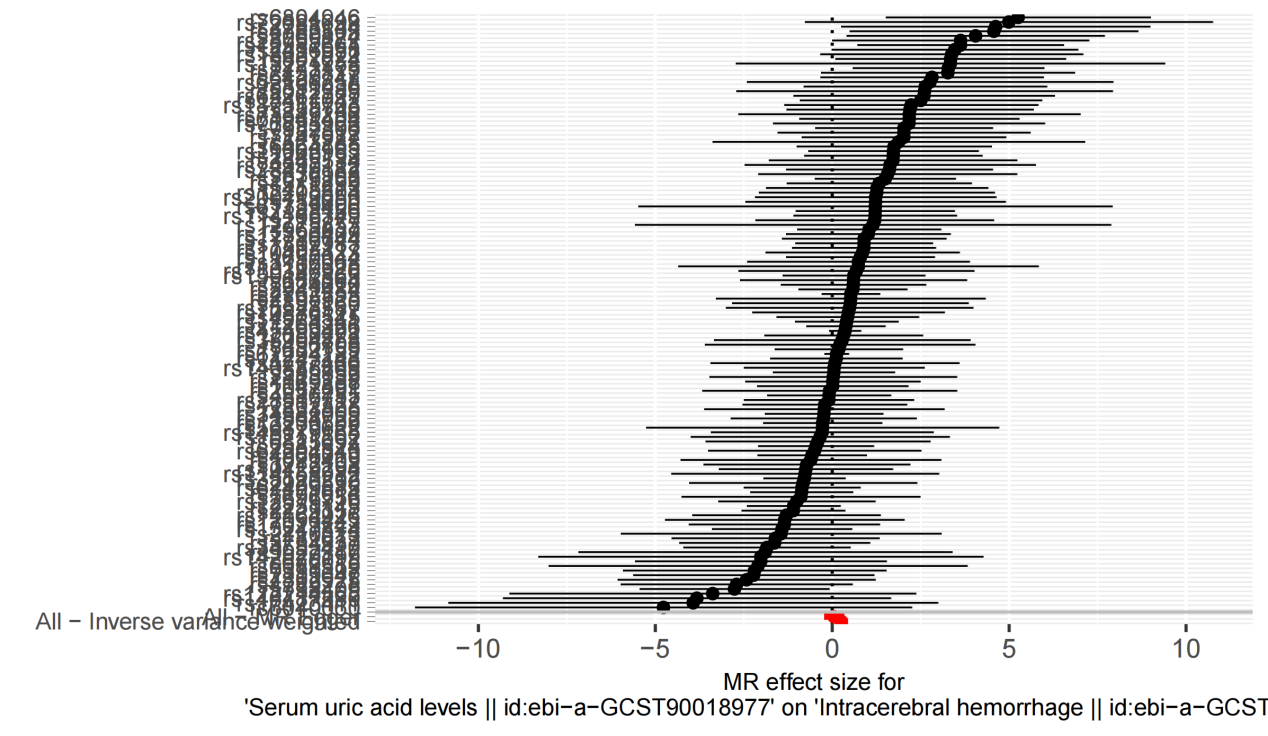


1. Serum uric acid and subarachnoid hemorrhage


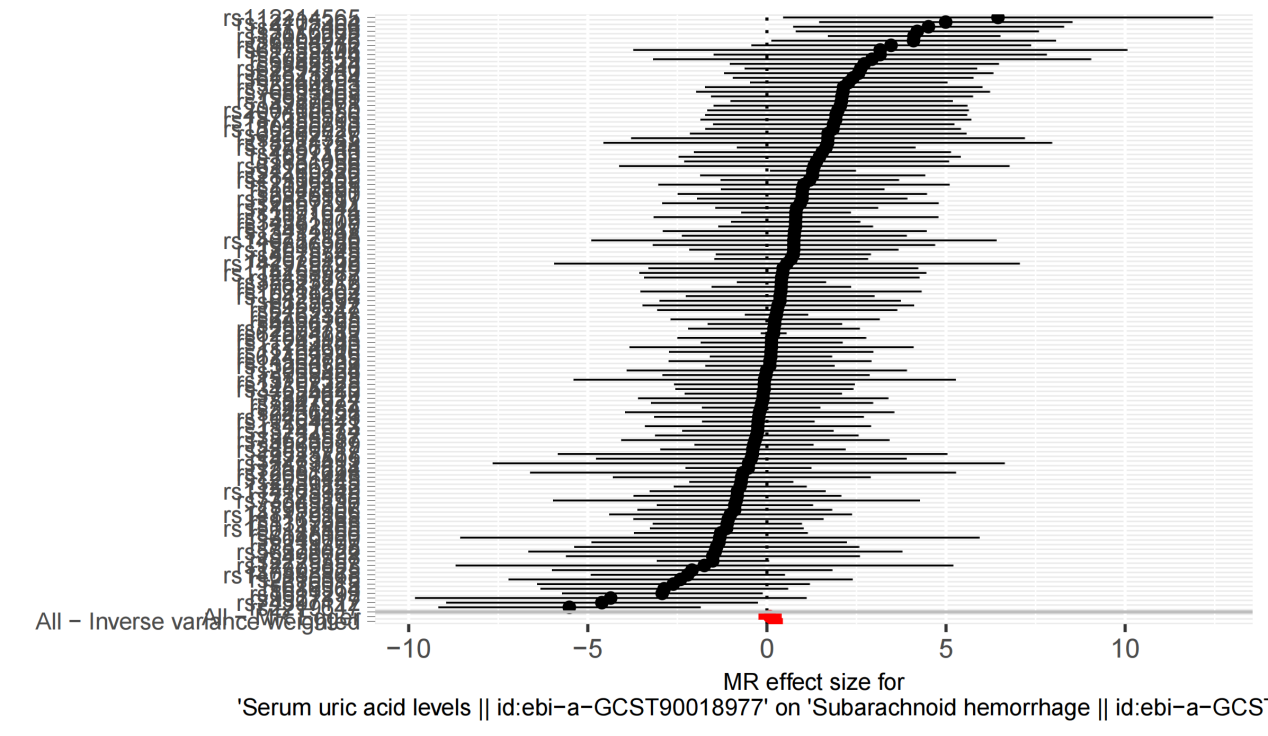


Figure S3 Funnel plot of causal associations

1. Serum uric acid and intracerebral hemorrhage


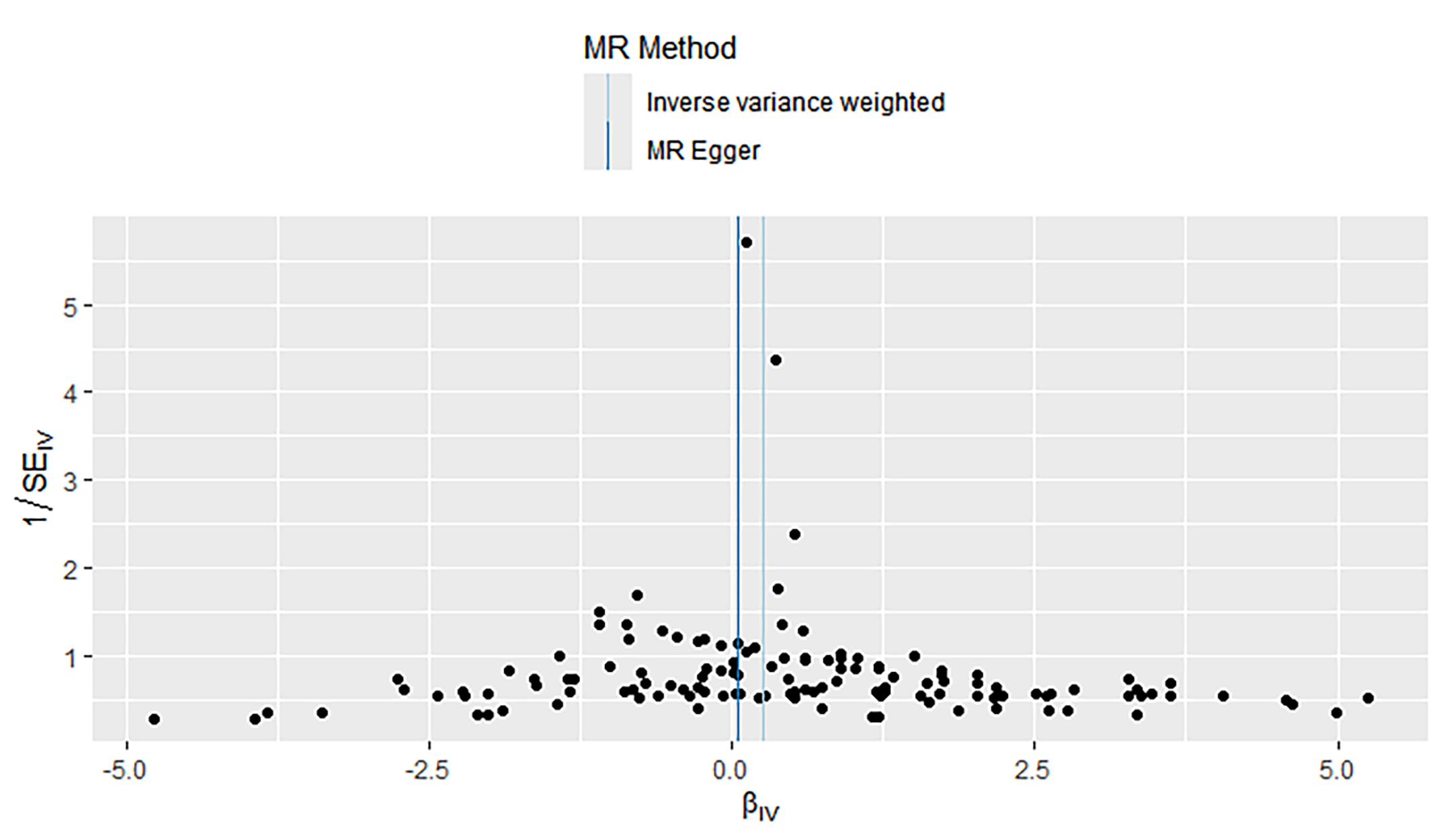


1. Serum uric acid and subarachnoid hemorrhage


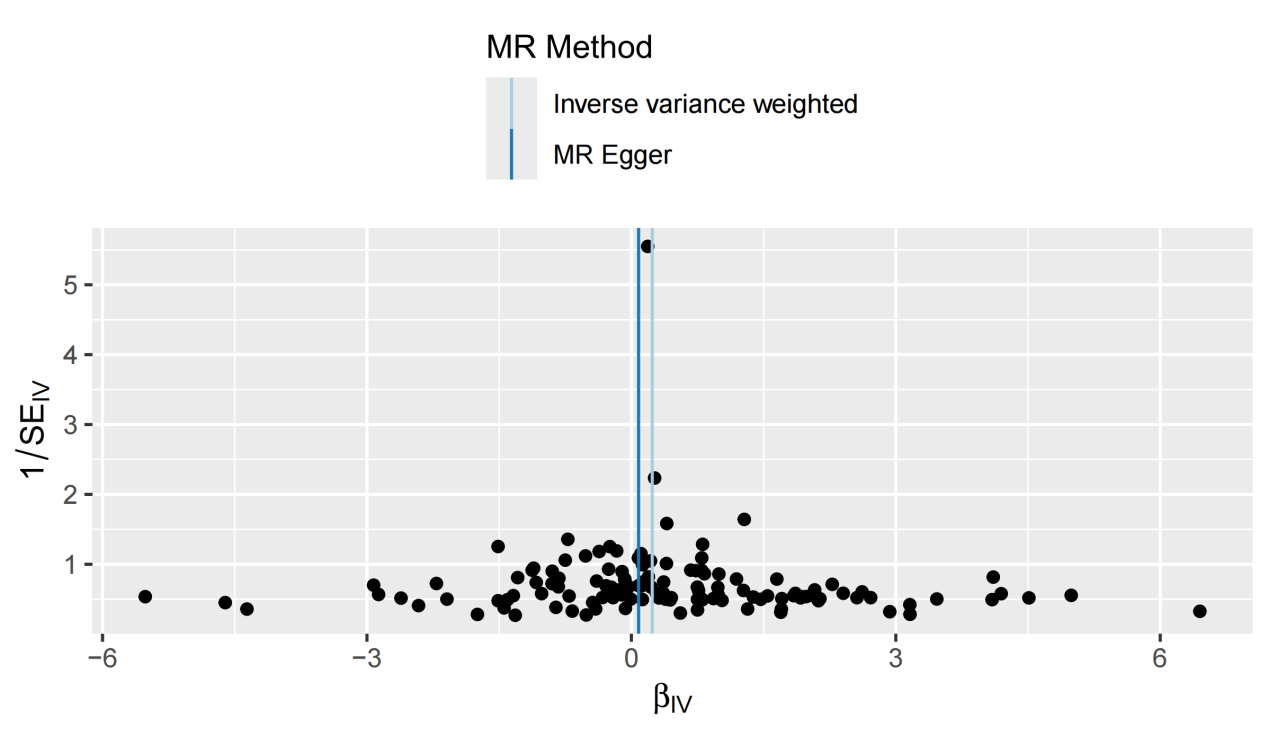


Figure S4 Leave-one-out plot to assess if a single variant is driving the association

1. Serum uric acid and intracerebral hemorrhage


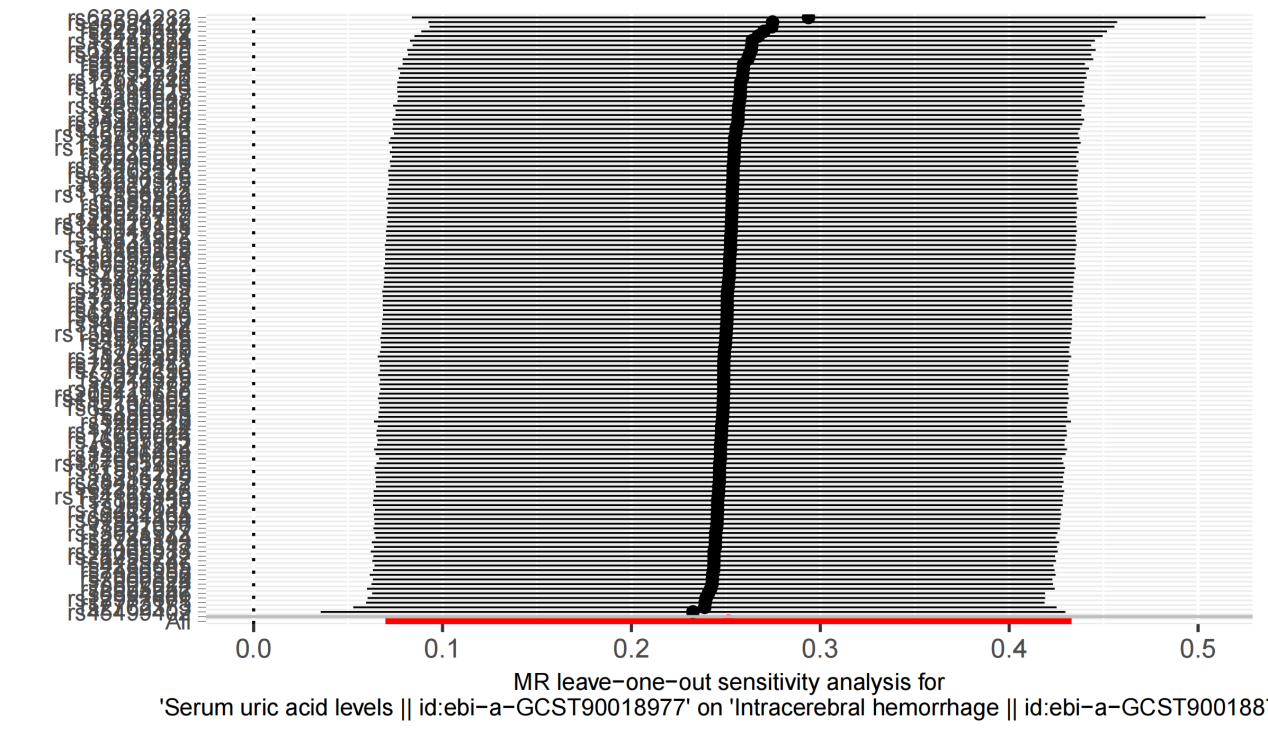


1. Serum uric acid and subarachnoid hemorrhage


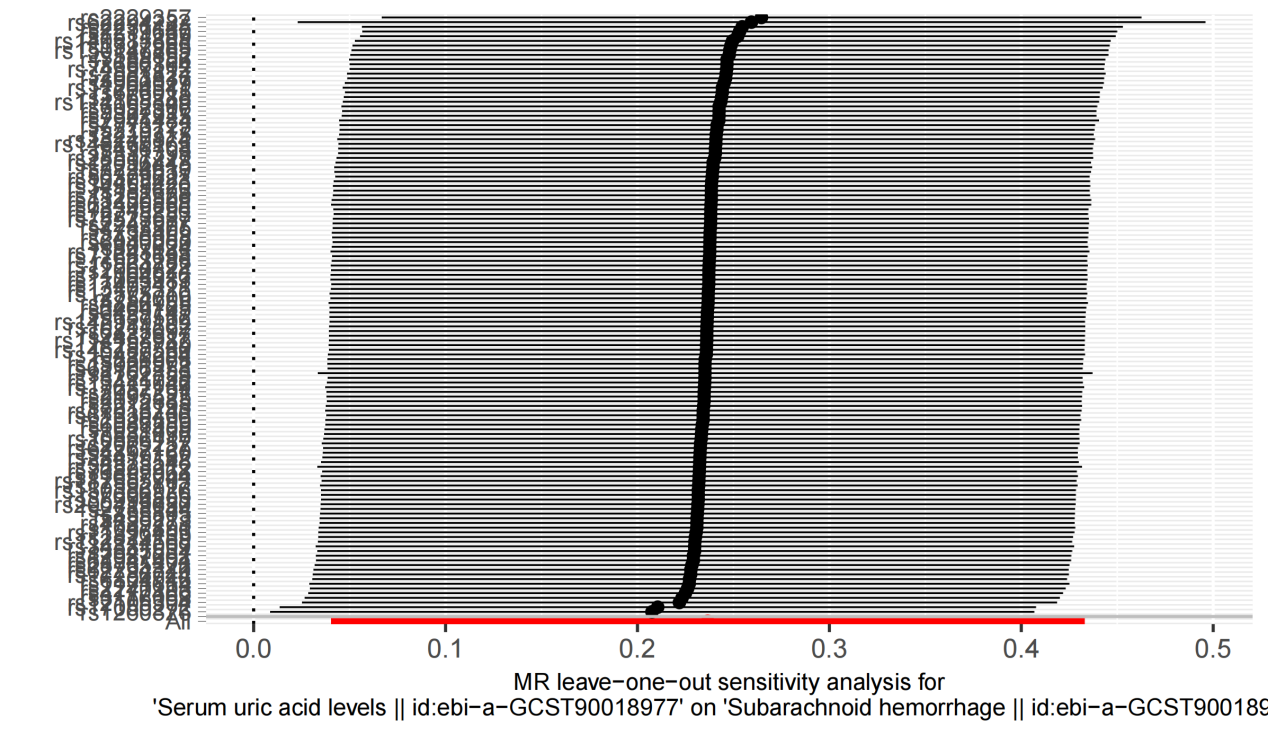

Supplement: Supplementary file 6 [file mmc6.docx]
